# Association of the use of nonfood prebiotics, probiotics, and synbiotics with total and cause-specific mortality: a prospective cohort study

**DOI:** 10.1186/s12937-025-01104-w

**Published:** 2025-03-20

**Authors:** Luyan Zheng, Jiaqi Zhang, Jing Yang, Yanbo Wang, Yina Zhang, Kailu Fang, Jie Wu, Min Zheng

**Affiliations:** 1 State Key Laboratory for Diagnosis and Treatment of Infectious Diseases, Collaborative Innovation Center for Diagnosis and Treatment of Infectious Diseases, The First Affiliated Hospital, College of Medicine, National Clinical Research Center for Infectious Diseases, Zhejiang University, #79 Qingchun Road, 310003 Hangzhou, Zhejiang Province China; 2 The Second School of Medicine, Wenzhou Medical University, Wenzhou, Zhejiang China

**Keywords:** Nonfood, Prebiotics, Probiotics, Synbiotics, Mortality

## Abstract

**Background:**

The use of nonfood prebiotics, probiotics, and synbiotics has approximately tripled in the last 20 years. It is necessary to examine the associations of these substances with all-cause and cause-specific mortality in a large prospective cohort.

**Methods:**

This study included 53,333 adults from the National Health and Nutrition Examination Survey 1999–2018. All participants answered questions on the use of dietary supplements and medications, including prebiotics, probiotics, and synbiotics. Death outcomes were determined by linkage to National Death Index records through 31 December 2019. Cox proportional hazards models were used to estimate hazard ratios (HR) and 95% confidence intervals (CI) for mortality from all causes, heart diseases, cancer, and other causes.

**Results:**

During a mean follow-up of 10.6 years, 9117 deaths were documented, including 2364 heart disease deaths, 1964 cancer deaths, and 4700 other causes deaths. Compared to nonusers, nonfood prebiotic, probiotic, and synbiotic users had a 59% (HR 0.41, 95% CI 0.30 to 0.56), 56% (HR 0.44, 95% CI 0.26 to 0.74), 49% (HR 0.51, 95% CI 0.31 to 0.83), and 64% (HR 0.36, 95% CI 0.23 to 0.59) for lower risk of all-cause, cancer, heart disease, and other causes mortality, respectively. Moreover, the inverse association of the use of prebiotics, probiotics, and synbiotics with mortality was stronger in female participants and participants without hypertension.

**Conclusion:**

The use of nonfood prebiotics, probiotics, and synbiotics is significantly associated with lower all-cause mortality, as well as deaths from heart disease, cancer, and other causes.

**Supplementary Information:**

The online version contains supplementary material available at 10.1186/s12937-025-01104-w.

## Graphical Abstract

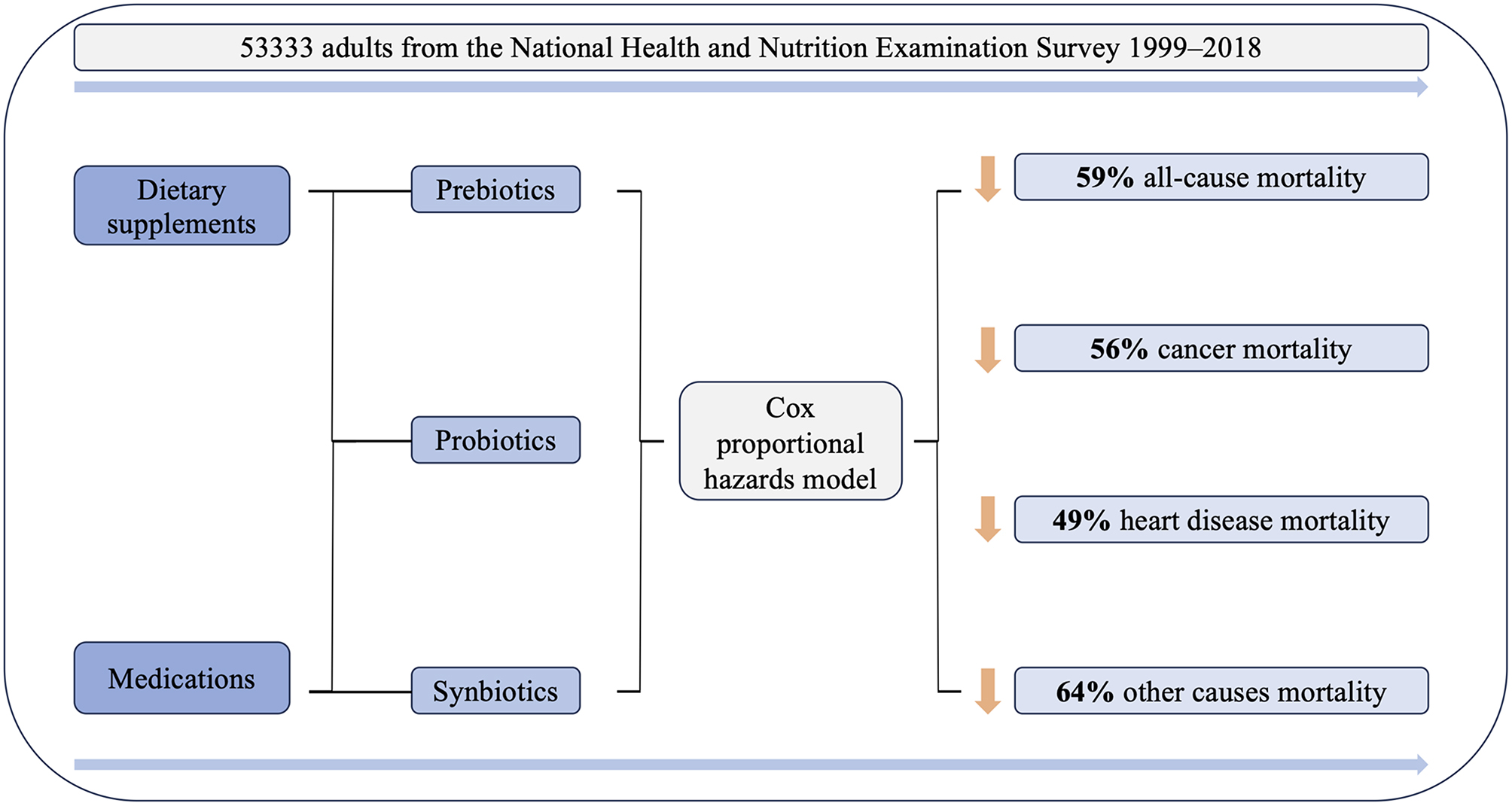

## Introduction

Prebiotics, probiotics, and synbiotics are attracting growing interest for their roles in regulating the gut microbiota as well as their anti-inflammatory and antioxidant properties [1, 2]. Prebiotics are substrates that are selectively utilized by host microorganisms conferring a health benefit, such as bifidogenic and galacto-oligosaccharides (GOS) [3]. Probiotics are live microorganisms with health benefits for the host [4]. Some probiotics, such as bifidobacterium and lactobacillus, are significant in the prevention of cardiovascular disease and cancer [5, 6]. Synbiotics are combinations of prebiotics and probiotics that enhance the viability of probiotic bacteria and facilitate more effective colon implantation [7]. High-fiber food (e.g., fruits and vegetables) and yogurt are natural sources of prebiotics and probiotics. The use of nonfood prebiotics, probiotics, and synbiotics has approximately tripled in the last 20 years due to growing research linking their usage to positive changes in gut microbiota and various clinical outcomes [8].

Nutrients obtained from food and supplements may confer different health effects. For example, a recent prospective cohort study has suggested that adequate intake of certain nutrients (such as vitamin A, vitamin K, and magnesium) is linked to a lower risk of death [9]. However, these associations are limited to nutrient intake from food rather than supplements. Several epidemiological studies have found an inverse association between the intake of prebiotics, probiotics, or synbiotics from food and mortality [10–13]. However, few studies have examined the impact of these substances from nonfood sources. Therefore, the associations between nonfood sources of prebiotics, probiotics, and synbiotics with mortality remain unclear.

As the use of nonfood prebiotics, probiotics, and synbiotics continues to grow as a preventative measure against various disorders, it becomes increasingly important to examine the relationship between the intake of these substances from nonfood sources and mortality, especially given the current lack of clear evidence. We hypothesized that nonfood sources of prebiotics, probiotics, and synbiotics may have a beneficial effect on mortality. The National Health and Nutrition Examination Survey (NHANES) is a nationally representative repeated cross-sectional survey of the US civilian population with extensive data on diets, dietary supplements, and linkage to health outcomes [14]. The main objective of this study was to examine the impacts of nonfood prebiotics, probiotics, and synbiotics on overall mortality as well as mortality related to specific causes through prospective analysis of data from NHANES.

## Methods

### Study population

**Fig. 1:**
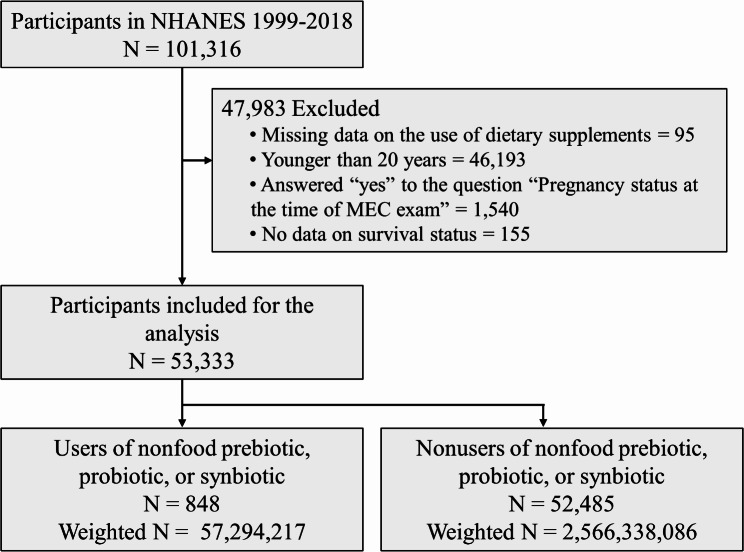
Flow diagram of study participant selection and exclusion. **Abbreviations**: NHANES, National Health and Nutrition Examination Survey; MEC, Mobile Examination Center

NHANES was carried out by the Centers for Disease Control and Prevention’s National Center for Health Statistics. The NHANES procedures were authorized by the Institutional Review Board of the National Center of Health Statistics [14]. Every participant in NHANES provided written consent after being fully informed. NHANES employed a complex and multi-stage probability sampling approach to select a sample that accurately represented the community-dwelling members of the US population. Details of the study design and data collection have been previously documented elsewhere [15, 16]. Participants from the continuous NHANES (1999–2018) datasets were initially included in the present analysis. Participants were then excluded based on the following exclusion criteria: no available data on the use of dietary supplements (*n* = 95), younger than 20 years old (*n* = 46 193), pregnant at the baseline survey (*n* = 1540), and no available mortality data (*n* = 155). For the current analysis, a total of 53,333 participants were ultimately included. Figure 1 shows the flowchart of cohort selection and exclusion.

### Assessments of nonfood prebiotic, probiotic, and synbiotic use

Data for prebiotic, probiotic, and synbiotic products are available in both dietary supplement questionnaires and prescription medication questionnaires. Details on the data extraction methods regarding nonfood prebiotic, probiotic, and synbiotic intake have been previously reported [8]. In brief, the interviewer-administered questionnaire conducted during the household interview phase of NHANES collected data on the use of dietary supplements and prescription medications within the last 30 days. This information encompassed details such as the names of the dietary supplements and medications, as well as their respective ingredients. The participants were asked by the interviewer about their consumption of dietary supplements (both prescribed and nonprescribed) or any prescription medications within the last 30 days. The interviewer also gathered data on how frequently, for how long, and in what quantities the supplements or medications were taken. The interviewer took thorough notes on each reported product, checked labeled containers if they were present, and entered the information from the product labels into the survey tablet. In cases where containers were not accessible, the participants were requested to provide verbal details about the products they had taken. The nutritionists at NHANES conducted text searches in order to locate the most similar entries in the NHANES Dietary Supplement Database or the NHANES prescription medication database. This was done to identify products that contained prebiotics, probiotics, and synbiotics [8, 17]. Finally, the duration of prebiotic, probiotic, and synbiotic use as well as the reason for use (self- administered or doctor-advised) were extracted from the questionnaire. For our analysis, those participants who answered “yes” to the question (“Have you used or taken any vitamins, minerals or other dietary supplements in the past month?”) and supplied certain products containing prebiotics, probiotics, or synbiotics were identified as users of nonfood prebiotic, probiotic, or symbiotic. Those who did not meet these criteria were categorized as non-users. Search terms of products that contained prebiotics, probiotics, and synbiotics are displayed in the Supplementary Sect. 1.

### Assessments of prebiotic and probiotic use from food

Numerous foods, such as fruits, vegetables, and whole grains, are rich in dietary fiber, which is a major source of natural prebiotics [18]. The researchers gathered information on the consumption of dietary fiber by using 24-hour dietary recalls. These recalls were administered by skilled interviewers at NHANES Mobile Examination Centers, using computer-assisted interviews. Participants were first asked to recall all food and beverages consumed the day before the interview, including the frequency and quantity of intake (recorded in grams). The interview data files were subsequently transferred to a food coding and data management system created by the United States Department of Agriculture (USDA) [19]. Next, the amounts of energy and nutrient components, including dietary fiber from each food and beverage, were calculated using the USDA 1994–98 Survey Nutrient Database (1999–2000) and USDA Food and Nutrient Databases for Dietary Studies (FNDDS) (2001–2018) [20, 21]. In addition, yogurt, the main source of probiotics from food consumption, was also extracted from 24-hour dietary recalls [18]. We categorized yogurt consumption into the following three groups: a nonconsumption group (0 g/d), low-consumption group (≤ the sex-specific median intake), and high consumption group (> the sex-specific median intake) [18].

### Assessments of demographic and lifestyle factors and comorbidity conditions

During household interviews, standardized questionnaires were utilized to gather a range of information regarding demographics, lifestyle, and comorbidities. This included details such as age, gender, ethnicity, marital status, level of education, family income-to-poverty ratio (FIPR), body mass index (BMI), smoking and drinking habits, physical activity levels, self-reported health status, family history of diabetes or heart attack, presence of chronic diseases as self-reported, and dietary intake data obtained through one or two 24-hour dietary recalls. These data were preprocessed according to prior studies [22–25]. Race/ethnicity was categorized into non-Hispanic White, non-Hispanic Black, Mexican American, or other groups. Marital status was classified as married, separated (including widowed and divorced individuals), or never married. Further details on preprocessing are provided in the Supplementary Sect. 2.

Moreover, the evaluation of dietary quality was conducted using the Healthy Eating Index-2015 (HEI-2015) score. This score assesses how well individuals adhere to the important recommendations outlined in the 2015–2020 Dietary Guidelines for Americans [26]. The total HEI-2015 score ranges from 0 to 100, and a higher score indicates a healthier diet. Finally, prescription medication use, mainly including gastrointestinal and anti-infective agents, was also extracted, which has been demonstrated to influence gut microbiome composition and function [27, 28].

### Ascertainment of outcomes

We determined mortality status through linkage to the National Death Index through December 31, 2019 using a unique study identifier [29]. More information about the linkage method is provided in the National Center for Health Statistics [29]. We also ascertained causes of death according ICD-10 (International Classification of Diseases, 10th revision) codes. The study focused on identifying deaths caused by cancer by examining the underlying cause of death listed as the ICD-10 codes C00-C09. The primary results of the research encompassed mortality rates associated with various factors, including all causes of death, heart diseases (ICD-10 codes I00-I09, I11, I13, and I20-I51), cancer (ICD-10 codes C00-C97), and other causes.

### Statistical analysis

All analyses took into account the NHANES complex survey sampling design, incorporating sample weights, clustering, and stratification [30]. Percentage of missing data in the whole covariates was less than 5%. Missing values for any covariates were imputed by multiple imputation. The present analysis involved four main steps. First, we conducted a comparative analysis between individuals who consumed nonfood prebiotics, probiotics, or synbiotics and those who did not. The comparison involved examining the distribution of demographic factors, lifestyle factors, and comorbidity conditions. For continuous variables, t tests were used, while chi-square tests were employed for categorical variables. Subsequently, the Cox proportional hazards model was employed to assess the association between the duration of intake of nonfood prebiotics, probiotics, and synbiotics, and both all-cause and cause-specific mortality. The model provided hazard ratios (HRs) and 95% confidence intervals (CIs) as estimations of this association. In the present study, the following three multivariable models were constructed: Model 1 was adjusted for age, sex, and race/ethnicity; Model 2 was additionally adjusted (from Model 1) for education level, marital status, FIPR, smoking status, drinking status, BMI, physical activity level, family history of diabetes and heart attack, self-reported general health, self-reported chronic diseases (diabetes, hypertension, congestive heart disease, and chronic kidney disease), and HEI-2015 scores, as those covariates may be mediators for the association of nonfood prebiotic, probiotic, and synbiotic use with mortality [18, 23]; and Model 3 was additionally adjusted (from Model 2) for dietary fiber and yogurt consumption because they were the major sources of prebiotics and probiotics from food [18].

In order to examine how well the findings can be applied to various groups and to explore potential differences within those groups, we conducted subgroup analyses by the following stratifications: age (< 60 and ≥ 60 years old); gender (male and female); reasons for nonfood prebiotic, probiotic, and synbiotic intake (self- administered or doctor-advised); lifestyle habits (smoking and drinking status); disease status (diabetes and hypertension); BMI categories; physical activity levels; HEI-2015 scores; yogurt intake; and dietary fiber intake. Finally, we conducted several sensitivity analyses to ensure the reliability of our findings, including (1) eliminating participants who had incomplete data, (2) excluding individuals with a follow-up period of less than two years, (3) removing participants with cardiovascular disease or cancer, (4) excluding participants who were using gastrointestinal or anti-infective drugs, (5) removing participants whose duration of supplement use was less than half a year, and (6) assessing the association of prebiotic, probiotic, or synbiotic supplements with mortality as independent factors. Details of the sensitivity analyses are shown in the Supplementary Sect. 3. Python (version 3.9, Python Software Foundation) and R (version 4.1.2, R Foundation for Statistical Computing) were utilized for all analyses, and a two-tailed *P* value of < 0.05 was considered to be statistically significant.

## Results

### Population characteristics

Among the 53,333 participants, 20,586 (38.6%) reported the use of dietary supplements but only 848 (1.6%) reported the use of nonfood prebiotics, probiotics, or synbiotics within the past month. Among these users, 212 participants reported the use of nonfood prebiotics, 588 participants reported the use of nonfood probiotics, and 48 participants reported the use of nonfood synbiotics. Table 1 shows the baseline characteristics of the included participants from the NHANES dataset. Compared to nonusers, those who used nonfood prebiotics, probiotics, or synbiotics were older, more likely to be females, more likely to be non-Hispanic whites, have higher levels of education, have higher levels of family income, and be physically active. Compared to nonusers, those who used prebiotics, probiotics, and synbiotics also reported a higher prevalence of cancer at baseline (16.7% vs. 9.5%, *P* < 0.001). Moreover, prebiotic, probiotic, and synbiotic users had a healthier diet than nonusers, including higher HEI-2015 scores (57.0 vs. 51.3, *P* < 0.001) and higher dietary fiber intake (35.3 gm vs. 29.6 gm, *P* < 0.001).

**Table 1:** Characteristics of US adults by nonfood prebiotic, probiotic, and synbiotic use

Characteristics	Total adults	Users of nonfood prebiotic, probiotic, or synbiotic	Nonusers of nonfood prebiotic, probiotic, or synbiotic	*P*-value
Unweighted N	53,333	848	52,485	
Weighted N	2,623,632,303	57,294,217	2,566,338,086	
Age, years, mean (SE)	50.5 (0.1)	56.2 (0.6)	50.5 (0.1)	< 0.001
Female, n (%)	26,957, (50.5)	539 (63.6)	26,418 (50.3)	< 0.001
Race/Ethnicity, n (%)				< 0.001
Mexican American	9166 (17.2)	73 (8.6)	9093 (17.3)	
Non-Hispanic White	23,587 (44.2)	518 (61.1)	23,069 (44.0)	
Non-Hispanic Black	11,220 (21.0)	119 (14.0)	11,101 (21.2)	
Others	9360 (17.6)	138 (16.3)	9222 (17.6)	
Education level, n (%)				< 0.001
Less than high school	14,750 (27.7)	89 (10.5)	14,661 (27.9)	
High school or equivalent	12,391 (23.2)	148 (17.5)	12,243 (23.3)	
College or above	26,192 (49.1)	611 (72.1)	25,581 (48.7)	
Physical activity, n (%)				< 0.001
0 times/week	25,518 (47.8)	301 (35.5)	25,217 (48.0)	
1–2 times/week	8391 (15.7)	105 (12.4)	8286 (15.8)	
>=3 times/week	19,424 (36.4)	442 (52.1)	18,982 (36.2)	
Family Income to Poverty Ratio, n (%)				< 0.001
<=1	10,897 (20.4)	62 (7.3)	10,835 (20.6)	
1–3	22,913 (43.0)	325 (38.3)	22,588 (43.0)	
> 3	19,523 (36.6)	461 (54.4)	19,062 (36.3)	
Smoking status, n (%)				0.347
Yes	24,531 (46.0)	376 (44.3)	24,155 (46.0)	
No	28,802 (54.0)	472 (55.7)	28,330 (54.0)	
Drinking status, n (%)				0.089
Yes	31,787 (59.6)	530 (62.5)	31,257 (59.6)	
No	21,546 (40.4)	318 (37.5)	21,228 (40.4)	
BMI, n (%)				0.282
< 25	15,976 (30.0)	275 (32.4)	15,701 (29.9)	
25-29.9	18,089 (33.9)	279 (32.9)	17,810 (33.9)	
>=30	19,268 (36.1)	294 (34.7)	18,974 (36.2)	
Comorbidities, n (%)				
Hypertension	18,959 (35.5)	317 (37.4)	18,642 (35.5)	0.276
Diabetes*	7728 (14.5)	135 (15.9)	7593 (14.5)	0.253
Congestive heart failure	1899 (3.6)	24 (2.8)	1875 (3.6)	0.287
Cancer	5132 (9.6)	142 (16.7)	4990 (9.5)	< 0.001
Kidney diseases	1804 (3.4)	32 (3.8)	1772 (3.4)	0.59
HEI-2015, mean (SE)	51.4 (0.1)	57.0 (0.5)	51.3 (0.1)	< 0.001
HEI-2015, n (%)				< 0.001
Quarter 1	13,335 (25.0)	122 (14.4)	13,213 (25.2)	
Quarter 2	13,332 (25.0)	182 (21.5)	13,150 (25.1)	
Quarter 3	13,333 (25.0)	189 (22.3)	13,144 (25.0)	
Quarter 4	13,333 (25.0)	355 (41.9)	12,978 (24.7)	
Dietary fiber, gm, mean (SE)	29.7 (0.1)	35.3 (0.6)	29.6 (0.1)	< 0.001
Dietary fiber, n (%)*				< 0.001
Quarter 1	13,109 (24.5)	216 (25.5)	12,893 (24.6)	
Quarter 2	13,757 (25.9)	156 (18.4)	13,601 (25.9)	
Quarter 3	13,261 (24.8)	202 (23.8)	13,059 (24.9)	
Quarter 4	13,206 (24.7)	274 (32.3)	12,932 (24.6)	
Yogurt consumption, g, mean (SE)	213.4 (0.6)	211.7 (5.0)	213.5 (0.6)	0.881
Yogurt consumption, n (%)†				< 0.001
No consumption	48,581 (91.1)	695 (82.0)	47,886 (91.2)	
Low	2421 (4.5)	89 (10.5)	2332 (4.4)	
High	2331 (4.4)	64 (7.5)	2267 (4.3)	

All estimates accounted for complex survey designs. Continuous variables were expressed as mean (standard error, [SE]). Categorical variables were expressed as number (percentage).

*Dietary fiber intake was categorized by quarters.

†Yogurt consumption was defined as no consumption (0 g/d), low (≤ sex-specific median intake), and high (> sex-specific median intake).

**Abbreviations**: SE, standard error; BMI, body mass index; HEI, healthy eating index

### Associations of nonfood prebiotic, probiotic, and synbiotic intake with mortality

During a mean follow-up of 10.6 years, a total of 9117 deaths were observed, including 2364 heart disease deaths, 1964 cancer deaths, and 4700 other causes deaths. Among 848 participants who reported the use of nonfood prebiotics, probiotics, or synbiotics, the median (IQR) duration of use was 2.1 (0.25-4.00) years. Approximately 25% of users stated that they started using prebiotic, probiotic, or synbiotic after receiving a suggestion from a medical professional or healthcare provider. Table 2 shows the association of prebiotic, probiotic, and synbiotic use with mortality. After adjusting for age, sex, race/ethnicity, education level, marital status, BMI, family income, smoking status, drinking status, physical activity level, family history of diseases, self-reported general health, disease status, and HEI-2015 scores, we found significant inverse associations of nonfood prebiotic, probiotic, and synbiotic use with the risk of all-cause mortality, heart disease mortality, cancer mortality, and other causes of mortality (all *P* < 0.05). The results of the multivariate analysis showed that the use of nonfood prebiotics, probiotics, and synbiotics is linked to a reduced risk of death from any cause (HR 0.38, 95% CI 0.28 to 0.52), death from heart disease (HR 0.40, 95% CI 0.24 to 0.67), death from cancer (HR 0.49, 95% CI 0.30 to 0.80), and death from other causes (HR 0.34, 95% CI 0.21 to 0.54). We observed similar outcomes when we further adjusted for fiber and yogurt consumption (prebiotics and probiotics from food) with an HR of 0.41 (95% CI 0.30 to 0.56) for all-cause mortality, 0.44 (95% CI 0.26 to 0.74) for heart disease mortality, 0.51 (95% CI 0.31 to 0.83) for cancer mortality, and 0.36 (95% CI 0.23 to 0.59) for other causes of mortality.

**Table 2:** Hazard ratios (95% CIs) of mortality with prebiotic, probiotic, and synbiotic use in NHANES 1999–2018

Cause of death		Nonusers of nonfood prebiotic, probiotic, or synbiotic	Users of nonfood prebiotic, probiotic, or synbiotic	*P* value
All causes				
	Number of deaths/total	9028/52,485	89/848	
	Model 1*	1.00	0.30 (0.22 to 0.41)	< 0.001
	Model 2†	1.00	0.38 (0.28 to 0.52)	< 0.001
	Model 3‡	1.00	0.41 (0.30 to 0.56)	< 0.001
Heart diseases				
	Number of deaths	2364	25	
	Model 1*	1.00	0.29 (0.17 to 0.49)	< 0.001
	Model 2†	1.00	0.40 (0.24 to 0.67)	< 0.001
	Model 3‡	1.00	0.44 (0.26 to 0.74)	0.002
Cancer				
	Number of deaths	1964	28	
	Model 1*	1.00	0.42 (0.26 to 0.68)	< 0.001
	Model 2†	1.00	0.49 (0.30 to 0.80)	0.004
	Model 3‡	1.00	0.51 (0.31 to 0.83)	0.007
Other cause				
	Number of deaths	4700	36	
	Model 1*	1.00	0.26 (0.16 to 0.42)	< 0.001
	Model 2†	1.00	0.34 (0.21 to 0.54)	< 0.001
	Model 3‡	1.00	0.36 (0.23 to 0.59)	< 0.001

Complex survey designs are considered for all estimates.

*Model 1: adjusted for age, sex, and race/ethnicity.

†Model 2: further adjusted (from Model 1) for education level, marital status, family income-poverty ratio, smoking and drinking status, body mass index, physical activity level, family history of diabetes and heart attack, self-reported general health, healthy eating index scores, self-reported chronic diseases (diabetes, hypertension, congestive heart disease, chronic kidney disease).

‡Model 3: further adjusted (from Model 2) for fiber and yogurt consumption.

**Abbreviation**: CI, confident interval

### Subgroup and sensitivity analyses

**Fig. 2:**
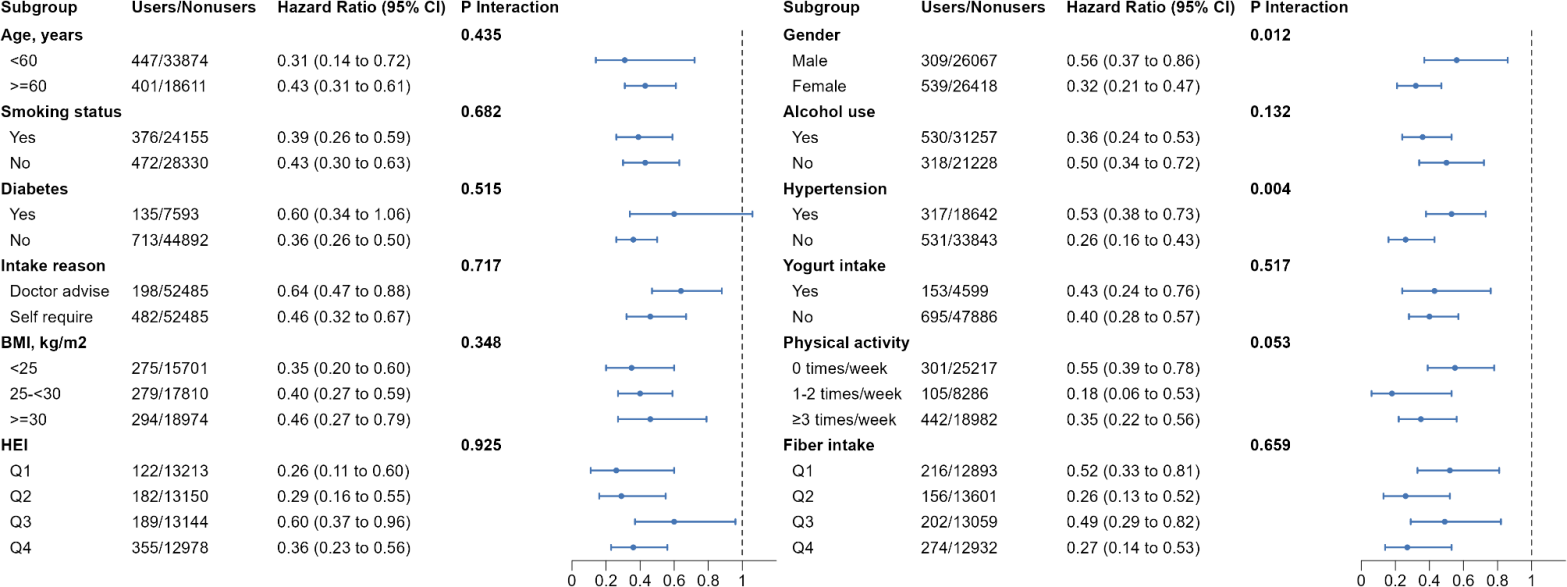
Subgroup analyses of the associations of prebiotic, probiotic, and synbiotic use with all-cause mortality risk (users vs. nonusers). All estimates accounted for complex survey design of NHANES. Risk estimates were adjusted for baseline age (not adjusted in subgroup analysis by age), sex (not adjusted in subgroup analysis by sex), race/ethnicity, education level, marital status, family income-poverty ratio, smoking status (not adjusted in subgroup analysis by smoking status), drinking status (not adjusted in subgroup analysis by drinking status), BMI (not adjusted in subgroup analysis by BMI), physical activity level (not adjusted in subgroup analysis by physical activity), family history of diabetes and heart attack, self-reported general health, HEI (not adjusted in subgroup analysis by HEI), self-reported chronic diseases (not adjusted in subgroup analysis by diabetes or hypertension), fiber intake (not adjusted in subgroup analysis by fiber intake), and yogurt consumption (not adjusted in subgroup analysis by yogurt consumption). **Abbreviations**: CI, confident interval; BMI, body mass index; HEI, healthy eating index

Figure 2 shows the stratified analyses of the associations of nonfood prebiotic, probiotic, and synbiotic use with all-cause mortality. In addition to gender and hypertension status, no significant interactions were detected for nonfood prebiotic, probiotic, and synbiotic use with the rest of the stratifying variables (P_interaction_ > 0.05). However, gender and hypertension status may modify the association of nonfood prebiotic, probiotic, and synbiotic intake with mortality. The inverse association between fiber intake and the outcome was found to be stronger among female participants compared to male participants. The hazard ratio (HR) for females was 0.32 (95% CI 0.21 to 0.47), while for males it was 0.56 (95% CI 0.37 to 0.86). The statistical analysis indicated a significant interaction (P_interaction_ = 0.012) between gender and the effect of fiber intake. The inverse association of nonfood prebiotic, probiotic, and synbiotic use with mortality was more evident among participants without hypertension than among those with hypertension (HR 0.26, 95% CI 0.16 to 0.43 vs. HR 0.53, 95% CI 0.38 to 0.73; P_interaction_ = 0.004). Furthermore, the inverse association between these supplements and mortality remained significant when evaluating the relationship between the consumption of non-food prebiotics, probiotics, and synbiotics with mortality, respectively. Supplementary Tables 1–6 show the results of the sensitivity analyses of the primary outcomes. There were no clear differences in any of the sensitivity analyses. A detailed description was shown in the Supplementary Sect. 4.

## Discussion

In the present large prospective cohort study, we analyzed data from 53,333 participants in the NHANES and found a significant inverse association of the use of nonfood prebiotics, probiotics, and synbiotics with all-cause, heart disease, cancer, and other causes of mortality after adjusting for a variety of confounders, including demographics, lifestyle factors, comorbidity conditions, and intake of prebiotics and probiotics from food. Specifically, compared to nonusers, individuals who used nonfood prebiotics, probiotics, or synbiotics had a significantly 59% lower risk of all-cause mortality, 56% lower heart disease mortality, 49% lower cancer mortality, and 64% lower other causes of mortality. Furthermore, the findings were strengthened through the inclusion of various stratified analyses and sensitivity analyses, enhancing their reliability. No significant interaction was observed for prebiotic, probiotic, and synbiotic use with most of the stratifying variables, except for gender and hypertension status.

Many previous studies have reported that the intake of prebiotics and probiotics from food is associated with lower mortality or a reduced risk of adverse health outcomes [18, 31–37]. For example, Liu et al. reported inverse associations of whole grain and dietary fiber intake with both incident liver cancer and chronic liver disease mortality among 485,717 retired participants [31]. In a pooled cohort of more than 1.44 million individuals, dietary fiber and yogurt consumption has been found to be significantly associated with a reduced risk of lung cancer after adjusting for known risk factors [18]. However, evidence linking the use of nonfood prebiotics, probiotics, and synbiotics to mortality is scarce. In addition, most previous studies have been conducted among specific populations, such as retired individuals [31], low-birth-weight infants [32], and individuals with health problems [33–37]. In the present study, we observed significant associations for the use of nonfood prebiotics, probiotics, and synbiotics with lower all-cause mortality and cause-specific mortality among the general population. The present findings may provide solid evidence for nonfood prebiotic, probiotic, and synbiotic recommendations for the general population.

Considering that the reasons for supplementing products may be associated with health status, we conducted a stratified analysis by intake reasons (self-administered or doctor-recommended) and found similar results in each stratum. Similarly, when further stratifying by lifestyle factors (including smoking, alcohol consumption, obesity, physical activity, and comorbidities), we obtained consistent results and did not find significant interactions. Of note, the inverse association of the intake of prebiotics, probiotics, and synbiotics with mortality was more pronounced in female participants than in males. The exact mechanism for this is still unclear, but mounting evidence suggests that estrogens may affect the gut microbiota, which further or synergistically significantly improves many health outcomes, especially for metabolic diseases [38, 39]. In addition, the association of prebiotic, probiotic, and synbiotic intake with all-cause mortality was slightly attenuated in participants with hypertension, suggesting that the development of hypertension is important for gut microbiota and dysbiosis [40]. Previous animal experiments have revealed that mice with high blood pressure experience an impaired intestinal barrier accompanied by heightened levels of inflammatory markers. However, when the blood pressure is reduced, the integrity of the intestinal barrier is restored [40–42]. Furthermore, sensitivity analyses demonstrated a strong negative association of prebiotic, probiotic, and synbiotic use with all-cause mortality after excluding participants who used anti-infective or gastrointestinal medications. Many antibiotics and gastrointestinal drugs have previously been shown to affect the intestinal microbiota and lead to disorders of the gut microbiota [27, 28, 43], which may weaken the beneficial effect of prebiotics, probiotics, and synbiotics.

Several mechanisms may explain the inverse associations of nonfood prebiotic and probiotic products with mortality. First, certain prebiotics and probiotics have the ability to individually or collaboratively influence the composition and activity of the gut microbiota, which then produces beneficial short-chain fatty acids regulating host immunity and metabolism, and these beneficial effects are not restricted to the gut but also affect various organs [44]. Two previous studies on older adults have demonstrated that daily consumption of galacto-oligosaccharide enhances phagocytic activity and natural killer cell activity [45, 46]. Second, prebiotics and probiotics restore microbial balance, thus inhibiting the proliferation of many harmful pathogens [44]. Third, studies performed in cell lines have shown that prebiotics and probiotics improve barrier function by upregulating the expression of tight junction proteins and mucus-secretion genes as well as downregulating inflammation [44, 47, 48]. Impaired barrier function allows translocation of inflammatory mediators from the intestine into the systemic circulation, which is known as metabolic endotoxemia and may also be a causative factor in many diseases [44, 49]. Fourth, prebiotics and probiotics may confer additive beneficial effect on long-term outcomes due to environmental factors. Environmental factors, especially microplastic toxicity, are clearly shown that play important roles in cardiovascular and cancer events via proinflammatory pathways [50]. Many nonfood probiotics contribute to the biodegradation of microplastic [51, 52]. Many randomized controlled trials have been conducted to investigate the efficacy of prebiotics, probiotics, and synbiotics in the treatment of human disease [53–55]. Nevertheless, further studies with larger sample sizes and longer time of follow-up are needed to assess the association of prebiotic, probiotic, or synbiotic treatments with other clinical outcomes.

### Strengths and limitations

To the best of our knowledge, this is the first prospective study that explores the associations of the use of nonfood prebiotics, probiotics, or symbioses with mortality. The strengths of this study include its design as a prospective study, a large sample size with long-term follow-up, and the utilization of a nationally representative sample from the general population, enabling better generalization of the findings. In addition, utilizing the extensive data obtained from the NHANES, we were able to conduct sensitivity and subgroup analyses as well as adjust for a wide range of potential confounding factors, such as socioeconomic status, comorbidities, dietary factors, and lifestyle factors. Nevertheless, there were several limitations in the present study. First, we were unable to determine causality due to the observational study design. Further randomized clinical trials that evaluate the health effects of nonfood prebiotics, probiotics, or symbioses are warranted. Second, the present study did not assess the dose‒response relationship of prebiotic, probiotic, and synbiotic use with mortality because the precise amount of dietary supplementary intake was not recorded with a uniform method. Third, the present study did not evaluate the association of specific prebiotic, probiotic, or synbiotic supplements with mortality as the sample size for a single product was too small for subgroup analysis. Finally, this study was unable to completely eliminate the possibility of confounding factors such as psychosocial stress, genetic vulnerability, or any other remaining or unidentified confounding variables.

## Conclusions

The present study showed that the utilization of nonfood prebiotics, probiotics, and synbiotics had a substantial association with reduced overall mortality and mortality linked to specific causes in a nationally representative sample, demonstrating the important role of nonfood prebiotics, probiotics, and synbiotics in the prevention of premature death. Although the results were robust in a series of stratified and sensitivity analyses, future clinical trials on the long-term health consequences of nonfood prebiotic, probiotic, and synbiotic use are still warranted.

## Electronic supplementary material

Below is the link to the electronic supplementary material.

Supplementary Material 1

## Data Availability

The datasets analyzed during the current study are available in https://www.cdc.gov/nchs/nhanes/index.htm.

## Abbreviations

GOS: Galacto-oligosaccharide
NHANES: National Health and Nutrition Examination Survey
USDA: United States Department of Agriculture
BMI: Body mass index
HEI: Healthy Eating Index
FIPR: Family income-to-poverty ratio
HRs: Hazard ratios
CIs: Confidence intervals
