# Supplementary material for: Association of the use of nonfood prebiotics, probiotics, and synbiotics with total and cause-specific mortality: a prospective cohort study
Source: Nutr J. 2025 Mar 20;24:45. doi: 10.1186/s12937-025-01104-w (PMC11924732; doi:10.1186/s12937-025-01104-w)
Supplement: Supplementary file 1 — Supplementary Material 1 [file 12937_2025_1104_MOESM1_ESM.docx]

**Association of the Use of Nonfood Prebiotics, Probiotics, and Synbiotics with Total and Cause-Specific Mortality: A Prospective Cohort Study**

**Contents**

[Supplementary Section 1. Search terms of prebiotic, probiotic, and synbiotic products 2](#_Toc190084536)

[Supplementary Section 2. Preprocessing of covariates 3](#_Toc190084537)

[Supplementary Section 3. Sensitivity analyses 4](#_Toc190084538)

[Supplementary Section 4. Results of the sensitivity analyses 5](#_Toc190084539)

[Supplementary Section 5. Supplementary Tables 6](#_Toc190084540)

[Supplementary Table 1. Hazard ratios (95% CIs) of mortality with prebiotic, probiotic, and synbiotic use after excluding participants with missing values. 6](#_Toc190084541)

[Supplementary Table 2. Hazard ratios (95% CIs) of mortality with prebiotic, probiotic, and synbiotic use after excluding participants who had follow-up time of less than two years. 7](#_Toc190084542)

[Supplementary Table 3. Hazard ratios (95% CIs) of mortality with prebiotic, probiotic, and synbiotic use after excluding participants with cardiovascular disease or cancer. 8](#_Toc190084543)

[Supplementary Table 4. Hazard ratios (95% CIs) of mortality with prebiotic, probiotic, and synbiotic use after excluding participants with the use of gastrointestinal or anti-infective drugs. 9](#_Toc190084544)

[Supplementary Table 5. Hazard ratios (95% CIs) of mortality with prebiotic, probiotic, and synbiotic use after excluding participants whose duration time of supplements use less than half year. 10](#_Toc190084545)

[Supplementary Table 6. Hazard ratios (95% CIs) of mortality with prebiotic, probiotic, and synbiotic use, separately. 11](#_Toc190084546)

[References: 12](#_Toc190084547)

# Supplementary Section 1. Search terms of prebiotic, probiotic, and synbiotic products

| Prebiotics |
| --- |
| ACADIA GUM, CHICOR, GLUCAN, GUM ARABIC, INULIN, LACTULOSE, OLIGOFRUC, OLIGOSAC, POLCYDEXTROSE, PREBIOTIC, PRE-BIOTIC, PREBIOTIC, PSYLLIUM, RESISTANT STARCH, WHEAT DEXTRIN |
| Probiotics |
| ACIDOPHILUS, ANIMALIS, BACILLUS, BACILLI, BIFIDOBACTERI, BIFIDUM, BOULARDII, BREVE, BREVIS, BUCHNERI, BULGARICUS, BUTYRICUM, CASEI ,CAUCASICUS, CEREVISIAE, CLAUSII, CLOSTRIDI, COAGULANS, CORYNIFORMIS, CRISPATUS, DELBRUECKII, ESCHERICH, E. COLI, ECOLI, E COLI, ENTEROCOCCUS, FAECALIS, FAECIUM, FERMENTUM, FLORENTINUS, GASSERI, HELVETICUS, INFANTIS, JOHNSONII, LACTIS, LACTIC ACID, BACTERIA, LACTOBACILL, LACTOCOCCUS, LEICHMANNII, LEUCONOSTOC, LICHENIFORMIS, LONGUM, MESENTERIC, MITIS, NISSLE, OLIGONITROPHILUS, ORALIS, PARACASEI, PEDIOCOCCUS, PLANTARUM, PROBIOTIC, PRO-BIOTIC, PRO BIOTIC, PROPIONIBACTERI, RATTUS, REUTERI, RHAMNOSUS, SACCHAROMYC, SALIVARIUS, SANGUIS, STEAROTHERMOPHILUS, STREPTOCOCCUS, SUBTILIS, THERMOPHILUS |
| Synbiotics |
| SYNBIOTIC, SYN-BIOTIC, SYN BIOTIC, as well as the combination of at least 1 prebiotic and 1 probiotic search term previously described or the dual use of a prebiotic product and a probiotic product |

# Supplementary Section 2. Preprocessing of covariates

Education level was divided into three categories: less than high school, high school or equivalent, and college or higher. Family income-to-poverty ratio (FIPR) was classified as <=1, 1–3, or >3. BMI was calculated by dividing weight in kilograms by height in meters squared, and it was categorized into three groups: normal weight (<25.0), overweight (25.0-29.9), and obesity (≥30.0). Smoking status was determined by the following question on the questionnaire: “Have you smoked at least 100 cigarettes in your life?”. Similarly, drinking status was determined by the following question on the questionnaire: “In any one year, have you had at least 12 drinks of any type of alcohol beverage?”. Moreover, information on moderate-to-vigorous physical activity during leisure-time and divided it into three distinct groups: 0, 1-2, and ≥3 times/week. The self-assessed health condition was divided into three categories: poor to fair, good, and very good to excellent. Baseline histories of diabetes, hypertension, congestive heart failure, cancer (excluding skin cancer), and chronic kidney diseases were also obtained as these comorbidities may be confounders for the association of nonfood prebiotics, probiotics, synbiotics intake with mortality ^(1,2)^.

# Supplementary Section 3. Sensitivity analyses

We conducted several sensitivity analyses to ensure the reliability of our findings. Initially, we eliminated participants who had incomplete data for covariates and conducted analyses using only complete cases. Secondly, we excluded individuals with a follow-up period of less than two years to minimize the potential bias of reverse causation. Thirdly, we removed participants with cardiovascular disease or cancer to mitigate the influence of severe illness on reverse causation. Fourthly, we excluded participants who were using gastrointestinal or anti-infective drugs to minimize the impact of inconsistent gut microbiota caused by these medications when evaluating the effects of the intervention ^(3)^. Next, we excluded participants whose duration of supplement use was less than half a year because some supplements require a longer period of time to change the human intestinal flora. Finally, we evaluated the associations between the consumption of non-food prebiotics, probiotics, and synbiotics with mortality, respectively.

# Supplementary Section 4. Results of the sensitivity analyses

Supplementary Tables 2-5 show the results of the sensitivity analyses of the primary outcomes. Sensitivity analyses showed no substantial change after the following exclusions: participants for whom covariate data were missing (Supplementary Table 2); those who had a follow-up time of less than two years (Supplementary Table 3); those with cardiovascular disease and cancer (Supplementary Table 4); and those for whom the duration of supplement use was less than half a year (Supplementary Table 5). The negative association of nonfood prebiotic, probiotic, and synbiotic use with mortality was more evident when participants with the use of gastrointestinal or anti-infective drugs were removed, resulting in HRs of 0.37 (95% CI 0.27 to 0.52) for all-cause mortality, 0.39 (95% CI 0.20 to 0.78) for heart disease mortality, 0.55 (95% CI 0.31 to 0.96) for cancer mortality, and 0.29 (95% CI 0.16 to 0.50) for other causes of mortality. After removing participants whose duration of use was less than half a year, an evident negative association (HR 0.40, 95% CI 0.27 to 0.60) of nonfood prebiotic, probiotic, and synbiotic use with all-cause mortality was also observed (Supplementary Table 5).

# Supplementary Section 5. Supplementary Tables

## Supplementary Table 1. Hazard ratios (95% CIs) of mortality with prebiotic, probiotic, and synbiotic use after excluding participants with missing values.

| **Cause of death** | | **Nonusers of nonfood prebiotic, probiotic, or synbiotic** | **Users of nonfood prebiotic, probiotic, or synbiotic** | **P value** |
| --- | --- | --- | --- | --- |
| All causes | |  |  |  |
|  | Number of deaths/total | 6205/36741 | 60/592 |  |
|  | Model 1* | 1.00 | 0.30 (0.21 to 0.43) | <0.001 |
|  | Model 2† | 1.00 | 0.29 (0.14 to 0.60) | <0.001 |
|  | Model 3‡ | 1.00 | 0.36 (0.18 to 0.72) | 0.004 |
| Heart diseases | |  |  |  |
|  | Number of deaths | 1906 | 17 |  |
|  | Model 1* | 1.00 | 0.26 (0.15 to 0.45) | <0.001 |
|  | Model 2† | 1.00 | 0.29 (0.17 to 0.49) | 0.001 |
|  | Model 3‡ | 1.00 | 0.34 (0.20 to 0.56) | 0.002 |
| Cancer | |  |  |  |
|  | Number of deaths | 1422 | 15 |  |
|  | Model 1* | 1.00 | 0.39 (0.22 to 0.65) | <0.001 |
|  | Model 2† | 1.00 | 0.41 (0.29 to 0.77) | <0.001 |
|  | Model 3‡ | 1.00 | 0.49 (0.30 to 0.81) | 0.007 |
| Other cause | |  |  |  |
|  | Number of deaths | 2877 | 28 |  |
|  | Model 1* | 1.00 | 0.35 (0.26 to 0.47) | <0.001 |
|  | Model 2† | 1.00 | 0.37 (0.23 to 0.64) | <0.001 |
|  | Model 3‡ | 1.00 | 0.44 (0.29 to 0.79) | <0.001 |
| Complex survey designs are considered for all estimates. *Model 1: adjusted for age, sex, and race/ethnicity.  †Model 2: further adjusted (from Model 1) for education level, marital status, family income-poverty ratio, smoking and drinking status, body mass index, physical activity level, family history of diabetes and heart attack, self-reported general health, healthy eating index scores, self-reported chronic diseases (diabetes, hypertension, congestive heart disease, chronic kidney diseases).  ‡Model 3: further adjusted (from Model 2) for fiber and yogurt consumption. **Abbreviation:** CI, confident interval. | | | | |

## Supplementary Table 2. Hazard ratios (95% CIs) of mortality with prebiotic, probiotic, and synbiotic use after excluding participants who had follow-up time of less than two years.

| **Cause of death** | | **Nonusers of nonfood prebiotic, probiotic, or synbiotic** | **Users of nonfood prebiotic, probiotic, or synbiotic** | **P value** |
| --- | --- | --- | --- | --- |
| All causes | |  |  |  |
|  | Number of deaths/total | 8894/47228 | 80/642 |  |
|  | Model 1* | 1.00 | 0.30 (0.21 to 0.41) | <0.001 |
|  | Model 2† | 1.00 | 0.36 (0.26 to 0.51) | <0.001 |
|  | Model 3‡ | 1.00 | 0.40 (0.28 to 0.55) | <0.001 |
| Heart diseases | |  |  |  |
|  | Number of deaths | 2329 | 24 |  |
|  | Model 1* | 1.00 | 0.32 (0.18 to 0.55) | <0.001 |
|  | Model 2† | 1.00 | 0.42 (0.24 to 0.72) | 0.002 |
|  | Model 3‡ | 1.00 | 0.46 (0.27 to 0.79) | 0.005 |
| Cancer | |  |  |  |
|  | Number of deaths | 1934 | 24 |  |
|  | Model 1* | 1.00 | 0.41 (0.24 to 0.70) | 0.001 |
|  | Model 2† | 1.00 | 0.46 (0.27 to 0.79) | 0.004 |
|  | Model 3‡ | 1.00 | 0.48 (0.28 to 0.83) | 0.008 |
| Other cause | |  |  |  |
|  | Number of deaths | 4631 | 32 |  |
|  | Model 1* | 1.00 | 0.25 (0.15 to 0.41) | <0.001 |
|  | Model 2† | 1.00 | 0.31 (0.19 to 0.51) | <0.001 |
|  | Model 3‡ | 1.00 | 0.34 (0.21 to 0.55) | <0.001 |
| Complex survey designs are considered for all estimates. *Model 1: adjusted for age, sex, and race/ethnicity.  †Model 2: further adjusted (from Model 1) for education level, marital status, family income-poverty ratio, smoking and drinking status, body mass index, physical activity level, family history of diabetes and heart attack, self-reported general health, healthy eating index scores, self-reported chronic diseases (diabetes, hypertension, congestive heart disease, chronic kidney diseases).  ‡Model 3: further adjusted (from Model 2) for fiber and yogurt consumption. **Abbreviation:** CI, confident interval. | | | | |

## Supplementary Table 3. Hazard ratios (95% CIs) of mortality with prebiotic, probiotic, and synbiotic use after excluding participants with cardiovascular disease or cancer.

| **Cause of death** | | **Nonusers of nonfood prebiotic, probiotic, or synbiotic** | **Users of nonfood prebiotic, probiotic, or synbiotic** | **P value** |
| --- | --- | --- | --- | --- |
| All causes | |  |  |  |
|  | Number of deaths/total | 6293/46016 | 55/691 |  |
|  | Model 1* | 1.00 | 0.24 (0.16 to 0.34) | <0.001 |
|  | Model 2† | 1.00 | 0.31 (0.22 to 0.45) | <0.001 |
|  | Model 3‡ | 1.00 | 0.33 (0.23 to 0.47) | <0.001 |
| Heart diseases | |  |  |  |
|  | Number of deaths | 1618 | 16 |  |
|  | Model 1* | 1.00 | 0.23 (0.11 to 0.49) | <0.001 |
|  | Model 2† | 1.00 | 0.33 (0.15 to 0.69) | 0.004 |
|  | Model 3‡ | 1.00 | 0.35 (0.16 to 0.75) | 0.007 |
| Cancer | |  |  |  |
|  | Number of deaths | 1258 | 18 |  |
|  | Model 1* | 1.00 | 0.44 (0.24 to 0.81) | 0.008 |
|  | Model 2† | 1.00 | 0.50 (0.27 to 0.93) | 0.029 |
|  | Model 3‡ | 1.00 | 0.52 (0.28 to 0.98) | 0.041 |
| Other cause | |  |  |  |
|  | Number of deaths | 3417 | 21 |  |
|  | Model 1* | 1.00 | 0.17 (0.10 to 0.30) | <0.001 |
|  | Model 2† | 1.00 | 0.23 (0.14 to 0.40) | <0.001 |
|  | Model 3‡ | 1.00 | 0.25 (0.14 to 0.42) | <0.001 |
| Complex survey designs are considered for all estimates. *Model 1: adjusted for age, sex, and race/ethnicity.  †Model 2: further adjusted (from Model 1) for education level, marital status, family income-poverty ratio, smoking and drinking status, body mass index, physical activity level, family history of diabetes and heart attack, self-reported general health, healthy eating index scores, self-reported chronic diseases (diabetes, chronic kidney diseases).  ‡Model 3: further adjusted (from Model 2) for fiber and yogurt consumption. **Abbreviation:** CI, confident interval. | | | | |

## Supplementary Table 4. Hazard ratios (95% CIs) of mortality with prebiotic, probiotic, and synbiotic use after excluding participants with the use of gastrointestinal or anti-infective drugs.

| **Cause of death** | | **Nonusers of nonfood prebiotic, probiotic, or synbiotic** | **Users of nonfood prebiotic, probiotic, or synbiotic** | **P value** |
| --- | --- | --- | --- | --- |
| All causes | |  |  |  |
|  | Number of deaths/total | 6793/44215 | 56/628 |  |
|  | Model 1* | 1.00 | 0.27 (0.19 to 0.38) | <0.001 |
|  | Model 2† | 1.00 | 0.35 (0.25 to 0.49) | <0.001 |
|  | Model 3‡ | 1.00 | 0.37 (0.27 to 0.52) | <0.001 |
| Heart diseases | |  |  |  |
|  | Number of deaths | 1782 | 14 |  |
|  | Model 1* | 1.00 | 0.26 (0.13 to 0.52) | <0.001 |
|  | Model 2† | 1.00 | 0.36 (0.18 to 0.72) | 0.004 |
|  | Model 3‡ | 1.00 | 0.39 (0.20 to 0.78) | 0.008 |
| Cancer | |  |  |  |
|  | Number of deaths | 1482 | 20 |  |
|  | Model 1* | 1.00 | 0.45 (0.25 to 0.79) | 0.005 |
|  | Model 2† | 1.00 | 0.53 (0.30 to 0.93) | 0.028 |
|  | Model 3‡ | 1.00 | 0.55 (0.31 to 0.96) | 0.037 |
| Other cause | |  |  |  |
|  | Number of deaths | 3529 | 22 |  |
|  | Model 1* | 1.00 | 0.20 (0.11 to 0.36) | <0.001 |
|  | Model 2† | 1.00 | 0.27 (0.15 to 0.47) | <0.001 |
|  | Model 3‡ | 1.00 | 0.29 (0.16 to 0.50) | <0.001 |
| Complex survey designs are considered for all estimates. *Model 1: adjusted for age, sex, and race/ethnicity.  †Model 2: further adjusted (from Model 1) for education level, marital status, family income-poverty ratio, smoking and drinking status, body mass index, physical activity level, family history of diabetes and heart attack, self-reported general health, healthy eating index scores, self-reported chronic diseases (diabetes, hypertension, congestive heart disease, chronic kidney diseases).  ‡Model 3: further adjusted (from Model 2) for fiber and yogurt consumption. **Abbreviation:** CI, confident interval. | | | | |

## Supplementary Table 5. Hazard ratios (95% CIs) of mortality with prebiotic, probiotic, and synbiotic use after excluding participants whose duration time of supplements use less than half year.

| **Cause of death** | | **Nonusers of nonfood prebiotic, probiotic, or synbiotic** | **Users of nonfood prebiotic, probiotic, or synbiotic** | **P value** |
| --- | --- | --- | --- | --- |
| All causes | |  |  |  |
|  | Number of deaths/total | 9028/52485 | 60/552 |  |
|  | Model 1* | 1.00 | 0.28 (0.19 to 0.42) | <0.001 |
|  | Model 2† | 1.00 | 0.36 (0.24 to 0.54) | <0.001 |
|  | Model 3‡ | 1.00 | 0.40 (0.27 to 0.60) | <0.001 |
| Heart diseases | |  |  |  |
|  | Number of deaths | 2364 | 17 |  |
|  | Model 1* | 1.00 | 0.29 (0.15 to 0.54) | <0.001 |
|  | Model 2† | 1.00 | 0.40 (0.21 to 0.74) | 0.004 |
|  | Model 3‡ | 1.00 | 0.45(0.24 to 0.84) | 0.012 |
| Cancer | |  |  |  |
|  | Number of deaths | 1964 | 19 |  |
|  | Model 1* | 1.00 | 0.40 (0.22 to 0.71) | 0.002 |
|  | Model 2† | 1.00 | 0.45 (0.25 to 0.81) | 0.008 |
|  | Model 3‡ | 1.00 | 0.48 (0.26 to 0.87) | 0.016 |
| Other cause | |  |  |  |
|  | Number of deaths | 4700 | 24 |  |
|  | Model 1* | 1.00 | 0.24 (0.13 to 0.45) | <0.001 |
|  | Model 2† | 1.00 | 0.32 (0.17 to 0.58) | <0.001 |
|  | Model 3‡ | 1.00 | 0.35 (0.19 to 0.65) | 0.001 |
| Complex survey designs are considered for all estimates. *Model 1: adjusted for age, sex, and race/ethnicity.  †Model 2: further adjusted (from Model 1) for education level, marital status, family income-poverty ratio, smoking and drinking status, body mass index, physical activity level, family history of diabetes and heart attack, self-reported general health, healthy eating index scores, self-reported chronic diseases (diabetes, hypertension, congestive heart disease, chronic kidney diseases).  ‡Model 3: further adjusted (from Model 2) for fiber and yogurt consumption. **Abbreviation:** CI, confident interval. | | | | |

## Supplementary Table 6. Hazard ratios (95% CIs) of mortality with prebiotic, probiotic, and synbiotic use, separately.

| **Cause of death** | | **Nonusers of nonfood prebiotic, probiotic, or synbiotic** | **Users of nonfood prebiotic, probiotic, or synbiotic** | **P value** |
| --- | --- | --- | --- | --- |
| Prebiotic users | |  |  |  |
|  | Number of deaths/total | 9028/52485 | 33/212 |  |
|  | Model 1* | 1.00 | 0.38 (0.25 to 0.60) | <0.001 |
|  | Model 2† | 1.00 | 0.48 (0.31 to 0.74) | 0.001 |
|  | Model 3‡ | 1.00 | 0.47 (0.29 to 0.74) | 0.001 |
| Probiotic users | |  |  |  |
|  | Number of deaths/total | 9028/52485 | 47/588 |  |
|  | Model 1* | 1.00 | 0.25 (0.16 to 0.37) | <0.001 |
|  | Model 2† | 1.00 | 0.32 (0.21 to 0.49) | <0.001 |
|  | Model 3‡ | 1.00 | 0.35 (0.23 to 0.53) | <0.001 |
| Due to the insufficient number of participants reporting the use of nonfood synbiotics (N = 48), the association between synbiotic consumption and mortality was not analyzed independently. Complex survey designs are considered for all estimates. *Model 1: adjusted for age, sex, and race/ethnicity.  †Model 2: further adjusted (from Model 1) for education level, marital status, family income-poverty ratio, smoking and drinking status, body mass index, physical activity level, family history of diabetes and heart attack, self-reported general health, healthy eating index scores, self-reported chronic diseases (diabetes, hypertension, congestive heart disease, chronic kidney disease).  ‡Model 3: further adjusted (from Model 2) for fiber and yogurt consumption. **Abbreviation:** CI, confident interval. | | | | |

# References:

1. Chen C, Ye Y, Zhang Y, et al. (2019) Weight change across adulthood in relation to all cause and cause specific mortality: prospective cohort study. *BMJ* **367**, l5584. England: .

2. Wan Z, Guo J, Pan A, et al. (2021) Association of Serum 25-Hydroxyvitamin D Concentrations With All-Cause and Cause-Specific Mortality Among Individuals With Diabetes. *Diabetes Care* **44**, 350–357. United States: .

3. Weersma RK, Zhernakova A & Fu J (2020) Interaction between drugs and the gut microbiome. *Gut* **69**, 1510–1519. England: .
